# Non-surgical therapy for the treatment of chronic low back pain in patients with Modic changes: A systematic review of the literature

**DOI:** 10.1016/j.heliyon.2022.e09658

**Published:** 2022-06-30

**Authors:** Xiaoping Mu, Wei Peng, Yufu Ou, Peifeng Li, Zhuhai Li, Jianxun Wei

**Affiliations:** a Department of Spine Surgery, The People's Hospital of Guangxi Zhuang Autonomous Region, Guangxi Academy of Medical Sciences, Nanning, China; b Institute of Anatomy and Cell Biology, Justus-Liebig-University, Giessen, Germany; c Department of Surgery, The University of Hong Kong-Shenzhen Hospital, Shenzhen, China

**Keywords:** Modic changes, Endplate signal changes, Non-surgical treatments, Systematic review

## Abstract

**Background:**

In absence of uniform therapeutic recommendations, knowledge of the available treatment options for Modic changes (MCs) patients and their safety and effectiveness would be crucial and significant for clinicians and such patients.

**Objectives:**

The aim of this study was to provide a systematic review of available studies on non-surgical treatments of MCs.

**Methods:**

We performed a systematic review of multiple electronic databases including PubMed, Web of Science, Embase, Cochrane Library, and China National Knowledge Infrastructure for the period until 31st August 2021 to search for studies on non-surgical treatments for MCs in accordance with the guidance of the Cochrane Handbook. Potential studies were screened by their titles and abstracts. The methodological quality of the included studies was independently evaluated by two authors. Final recommendations for the included interventions were developed based on grades of recommendations. The narrative format was adopted to synthesize the findings of the present work.

**Results:**

Fifth studies involving a total of 1147 patients were identified for this systematic review. The results of this review demonstrated that spinal manipulation has been suggested as an alternative option for patients with MCs. However, there was insufficient evidence to support that patients with MCs can benefit from the medication and wearing the rigid lumbar brace. Moreover, the rationale and safety for the use of antibiotics in such patients remain highly controversial. Low evidence revealed that exercise therapy might decrease pain intensity only for special subgroups of MCs patients.

**Conclusions:**

There is not yet enough evidence to suggest that non-surgical treatments are useful for patients with MCs. Further high-quality, multicenter trials are required to validate the effectiveness of these non-surgical treatments.

## 1 Introduction

Chronic low back pain (CLBP) is the leading health condition afflicting most of population in the context of population increases and ageing [1]. Due to its high rate of disability and health insurance use, CLBP has become a common disabling condition with adverse consequences worldwide [2, 3]. It has been reported that more than 80% of individuals will experience CLBP at least once at some point in their lifetime [4]. Therefore, CLBP exerts essential impacts not only on the individual but also adversely affects communities and health care systems [5].

It still remains unknown or uncertain about the etiology and pathomechanisms of CLBP. Osteoarthritis of the spine is widespread, with an estimated prevalence ranging from 40-85% [6]. Several studies [7, 8] have reported the relationship between CLBP and osteoarthritis degeneration of spine. Facet joint osteoarthritis itself has adequate nerve supply capable of leading low back pain [7]. The commonly accepted viewpoint in academia is that disc degeneration [9], facet joint arthrosis, and sacroiliac joint arthrosis [10] are the common causes of CLBP [11]. However, multiple conditions can affect facet and sacroiliac joint arthrosis, resulting in low back pain deriving from osteoarthritis degeneration of spine [11]. Moreover, osteoarthritis is the clinical outcome of a disease process that has been characterized by damage to articular cartilage, subchondral bone alteration, and a synovial inflammatory response [12], which may be strongly linked to the development of Modic changes (MCs) [8].

Signal intensity changes in the subchondral bone marrow adjacent to the vertebral endplate visible on magnetic imaging resonance (MRI), also known as MCs, have been the research hot on their clinical features and pathomechanisms. The histological manifestations and classifications of MCs were preliminarily elaborated by Modic et al. [13, 14] in 1988. It, coupled with their more severe radiographic performance than simple disc degeneration [15, 16] and positive correlation with CLBP [17], has also been attracted widespread academic attention. Although a recent paper from the Wakahama Spine Study [17], have reported a positive association between MCs and LBP, the most recent systematic review on this topic [18] concludes that the associations between MCs and LBP-related outcomes are inconsistent. Meanwhile, there are still no uniform therapeutic recommendations for MCs patients, and the establishment of treatment regimens depends primarily on the patient's symptoms (mainly CLBP). Therefore, non-surgical treatments such as physical therapy or medication are often recommended as the first-line treatment option for CLBP in clinical guidelines [19]. However, when the patient does not respond well to non-surgical treatments, then surgery should be considered.

Currently, a variety of non-surgical interventions targeting potential etiology and pathogenesis of MCs have been successively reported. However, the evidence on which non-surgical treatments are effective and safe for patients with MCs is inconclusive. The purpose of the present work was therefore to systematically review currently available studies on non-surgical treatments of MCs patients, with the aim of being able to find valuable evidence for treating such patients.

## 2 Materials and methods

This systematic review was carried out in strict accordance with the standard methodology of the Cochrane Handbook. The results of the study were reported under the guidance of the Preferred Reporting Items for Systematic Review and Meta-Analysis (PRISMA) proposed by the PRISMA Working Group [20]. The protocol of this review was registered with PROSPERO (registration number CRD42021272154). The present work is a secondary analysis of published studies on a specific topic. Therefore, ethics committee approval is not necessary for this study.

### 2.1 Search strategy

In the absence of international guidelines for the management of MCs, different therapeutic strategies have been described. It is therefore difficult to generalize these treatment options using several specific search terms. To perform a comprehensive search, it was decided after discussion in the research group to use the following search terms: “Modic changes”, “endplate signal changes”, “endplate bone marrow lesions”, “active discopathy”. Two reviewers with at least 3-year experience in literature retrieval independently searched electronic databases of Medline via PubMed, Web of Science, Embase, Cochrane Library, and China National Knowledge Infrastructure for the period until 31^st^ March 2022. Literature search strategy using PubMed database as an example is shown in File 1. No language limitation was settled for inclusion in the present work. We also manually searched the references of the included studies and relevant reviews to identify potential studies that were not retrieved in the preliminary search.

File 1 **Literature Search Strategy.**

PubMed – March 31st, 2022 – 1314 results

#1 Search: "Modic changes"[All Fields]

"Modic"[All Fields] AND ("change"[All Fields] OR "changed"[All Fields] OR "changes"[All Fields] OR "changing"[All Fields] OR "changings"[All Fields])

#2 Search: "endplate signal changes"[All Fields]

("endplate"[All Fields] OR "endplates"[All Fields]) AND ("signal transduction"[MeSH Terms] OR ("signal"[All Fields] AND "transduction"[All Fields]) OR "signal transduction"[All Fields] OR "signaling"[All Fields] OR "signal"[All Fields] OR "signal s"[All Fields] OR "signaled"[All Fields] OR "signaler"[All Fields] OR "signaler s"[All Fields] OR "signalers"[All Fields] OR "signalings"[All Fields] OR "signalization"[All Fields] OR "signalled"[All Fields] OR "signaller"[All Fields] OR "signaller s"[All Fields] OR "signallers"[All Fields] OR "signalling"[All Fields] OR "signallings"[All Fields] OR "signals"[All Fields]) AND ("change"[All Fields] OR "changed"[All Fields] OR "changes"[All Fields] OR "changing"[All Fields] OR "changings"[All Fields])

#3 Search: "endplate bone marrow lesions"[All Fields]

("endplate"[All Fields] OR "endplates"[All Fields]) AND ("bone marrow"[MeSH Terms] OR ("bone"[All Fields] AND "marrow"[All Fields]) OR "bone marrow"[All Fields]) AND ("lesion"[All Fields] OR "lesion s"[All Fields] OR "lesional"[All Fields] OR "lesions"[All Fields])

#4 Search: "active discopathy" [All Fields]

("activable"[All Fields] OR "activate"[All Fields] OR "activated"[All Fields] OR "activates"[All Fields] OR "activating"[All Fields] OR "activation"[All Fields] OR "activations"[All Fields] OR "activator"[All Fields] OR "activator s"[All Fields] OR "activators"[All Fields] OR "active"[All Fields] OR "actived"[All Fields] OR "actively"[All Fields] OR "actives"[All Fields] OR "activities"[All Fields] OR "activity s"[All Fields] OR "activitys"[All Fields] OR "motor activity"[MeSH Terms] OR ("motor"[All Fields] AND "activity"[All Fields]) OR "motor activity"[All Fields] OR "activity"[All Fields]) AND ("discopathies"[All Fields] OR "discopathy"[All Fields])

#5 Search: ("1988/01/01"[Date - Publication] : "2022/03/31"[Date - Publication])

(#1 OR #3 OR #3 OR #4) AND #5

Alt-text: File 1

### 2.2 Eligibility criteria

We developed the inclusion criteria for this systematic review based on the PICOs (population, intervention/exposure, comparison/control, outcome, and study design) principles of clinical interventional study: 1) Population: adult patients (age ≥18-year met the diagnostic criteria for CLBP (>6 months’ duration) [21] and had the evidence of MCs on lumbar MRI. The studies that included cases with a history of prior lumbar surgery would not be considered to include in the present work. 2) Intervention: medication and physical therapy. 3) Comparison: placebo, the standard of care, observation-only, or none. 4) Outcomes: any clinical outcomes including but not limited to pain intensity, disability, quality-of-life measures, the volume of MCs, or adverse events. 5) Study design: randomized controlled trial (RCT), observational cohort (single or double arms), or case series.

In this systematic review, we only included the one with the most complete outcome indicators for multiple papers from the same study. Review, animal experiment, case report, comment, and conference report were excluded. Moreover, the present work also excluded studies that did report results for a group of patients where some but not all had MCs, and for studies with interventions not specifically meant to target patients with MCs but where some had MCs.

### 2.3 Study selection

The retrieved records were all imported into EndNote, a world's essential reference management tool. Duplicate records from multiple databases were consolidated and then automatically eliminated. The titles and abstracts of the literature were independently browsed by two authors, marking these as included, excluded, or inconclusive. Studies marked as exclusion by both authors were preliminarily removed. Full texts of eligible and inconclusive studies were then downloaded and reviewed independently. The disagreement between authors was resolved by the consensus among researchers.

### 2.4 Data extraction

A standardized form was designed to summarize the characteristics and results of each included study. Information from the included studies was independently extracted by two authors and filled into the form. A third author was employed to check information in two forms from the above-mentioned authors. If necessary, the authors of the included studies were contacted by email for additional information about their studies. The following data items were extracted: 1) study characteristics: authors information, publication year, study design and place, follow-up, and population; 2) interventions: number of each group, and specific treatment options (dosage, duration); 3) outcomes measures and adverse events.

### 2.5 Risk of bias assessment

Two authors independently assessed the methodological quality of the included studies, and a third author was introduced to resolve disputes between the two authors. We adopted the bias risk tool (Table 1a) proposed by the Cochrane back review group to assess the risk of bias of the included RCTs [22], the Newcastle-Ottawa Quality Assessment Scale (NOQAS) [23] to evaluate the quality of the included comparative studies (Table 1b), and the evidence-based guideline development methodology of the North American Spine Society (NASS) to assess the level of evidence of the included case series (Table 1c).

**Table 1a:** Sources of risk of bias.

Bias Domain	Source of Bias	Answers
Selection	Was the method of randomization adequate?	Yes/No/Unsure
Selection	Was the treatment allocation concealed?	Yes/No/Unsure
Performance	Was the patient blinded to the intervention?	Yes/No/Unsure
Performance	Was the care provider blinded to the intervention?	Yes/No/Unsure
Detection	Was the outcome assessor blinded to the intervention?	Yes/No/Unsure
Attrition	Was the drop-out rate described and acceptable?	Yes/No/Unsure
Attrition	Were all randomized participants analyzed in the group to which they were allocated?	Yes/No/Unsure
Reporting	Are reports of the study free of suggestion of selective outcome reporting?	Yes/No/Unsure
Selection	Were the groups similar at baseline regarding the most important prognostic indicators?	Yes/No/Unsure
Performance	Were cointerventions avoided or similar?	Yes/No/Unsure
Performance	Was the compliance acceptable in all groups?	Yes/No/Unsure
Detection	Was the timing of the outcome assessment similar in all groups?	Yes/No/Unsure
Other	Are other sources of potential bias unlikely?	Yes/No/Unsure

**Table 1b:** Newcastle-Ottawa quality assessment scale (NOQAS).

Items	Descriptions
Selection (maximum 4 stars)	Is the case definition adequate
	Representativeness of the cases
	Selection of controls
	Definition of controls
Comparability (maximum 2 stars)	Study controls for the most important factor
	Study controls for any additional factor
Exposure (maximum 3 stars)	Ascertainment of exposure
	Same method of ascertainment for cases and controls
	Non-response rate

**Table 1c:** Levels of evidence for primary research question.

Level of Evidence	Details
Level I	• High quality randomized trial with statistically significant difference or no statistically significant difference but narrow confidence intervals • Systematic review of Level I RCTs (and study results were homogenous)
Level II	• Lesser quality RCT (eg, < 80% follow-up, no blinding, or improper randomization) • Prospective comparative study • Systematic review of Level II studies or Level I studies with inconsistent results
Level III	• Case-control study • Retrospective comparative study • Systematic review of Level III studies
Level IV	• Case series
Level V	• Expert opinion

Grades of recommendations for summaries or reviews of studies were used to assess the cumulative body of evidence for all identified interventions (Table 2). The strength of evidence across the studies was considered as one of the following four categories: good (mark as A), fair (B), poor (C), and insufficient or conflicting evidence (I).

**Table 2:** Grades of recommendations for summaries or reviews of studies.

Grade of Recommendation	Explanation	Standard Language	Levels of Evidence	
A	Good evidence (Level I studies with consistent findings) for or against recommending intervention.	Recommended	Two or more consistent Level I studies.	
B	Fair evidence (Level II or III studies with consistent findings) for or against recommending intervention.	Suggested	One Level I study with additional supporting Level II or III studies.	Two or more consistent level II or III studies.
C	Poor quality evidence (Level IV or V studies) for or against recommending intervention.	May be considered; is an option.	One Level I, II, III or IV study with supporting Level IV studies.	Two or more consistent Level IV studies.
I	There is insufficient or conflicting evidence not allowing a recommendation for or against intervention.	Insufficient evidence to make recommendation for or against.	A single level I, II, III or IV study without other supporting evidence.	More than one study with inconsistent findings∗.

∗ Note that in the presence of multiple consistent studies and a single outlying, inconsistent study, the Grade of Recommendation will be based on the level of the consistent studies.

## 3 Results

### 3.1 Literature search

**Figure 1:**
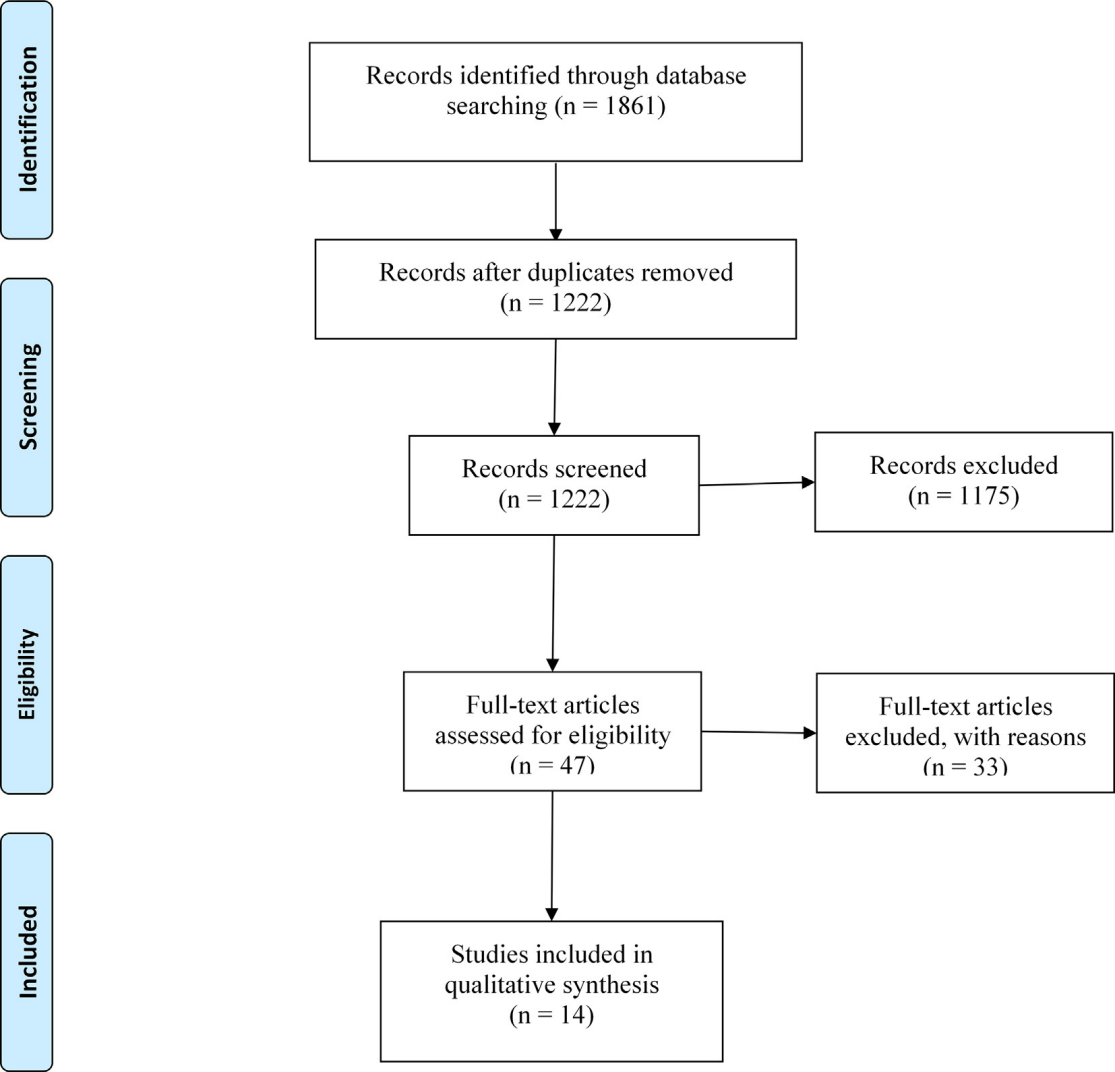
Flow diagram of study selection.

A flow chart of the literature retrieval process based on the PRISMA statement is presented in Figure 1. Our initial electronic search identified 2143 documents that potentially met the inclusion criteria under the established search strategy. After integration by EndNote software, 795 duplicated studies were excluded. Then, we eliminated 1348 irrelevant studies after a careful review of the titles and abstracts. The full texts of the remaining 54 papers were read, and the final 15 studies [24, 25, 26, 27, 28, 29, 30, 31, 32, 33, 34, 35, 36, 37, 38] that fully met the inclusion criteria were included in this systematic review.

### 3.2 Study characteristics and risk of bias

This systematic review included a total of 1147 patients in 15 studies involving a variety of non-surgical treatments. Nine studies targeted on the effects of medications on the patients with MCs, including antibiotics [26, 31, 32], glucosamine sulfate [33], zoledronic acid [27, 34, 35], calcitonin [36], and probiotics [37]. Additionally, 5 studies focused on the efficacy of spinal manipulation [28, 29], rigid lumbar brace [30] and exercise therapy [24, 25] in patients with MCs. The remaining one study [38] adopted a combination of physical therapy and medication. The characteristics and main findings of the included studies were summarized in Tables 3 and 4, respectively.

**Table 3:** Characteristics of the eligible trials.

Study, design, country, follow-up	Population characteristic		Eligibility criteria		Treatment	
	Intervention	Control	Inclusion	Exclusion	Intervention	Control
**Physiotherapy**						
Jensen RK et al. 2012, 2015; RCT; Denmark; 1 year.	N:51; mean age: 45; 69% female; median pain NRS: 5.1; mean disability RMQ- 23:13.3; mean general health EQ_VAS_ = 53.	N:49; mean age: 47; 67% female; median pain NRS:5.6; mean disability RMQ- 23:12.0; mean general health EQ_VAS_ = 54.	Adult patients with LBP or leg pain of at least 3 on an 11-point NRS; and duration of symptoms from 2 to 12 months; and M1, 2 or 3 with a distribution exceeding the endplate.	Patients with other physical or mental disorders; or a competing LBP etiology; or a history of spinal surgery with no pain relief after the operation.	Exercised in groups of up to a maximum of 10 people for one hour once a week for 10 weeks; and the same exercises at home three times a week.	Avoid hard physical activity and to rest twice daily for one hour for 10 weeks, by lying down.
Boutevillain L et al 2018; SC; France; 3 months.	N:62; mean age: 47; 34.5% female; baseline pain:6.7; night-time awakening due to pain:39; morning stiffness >15 min:41.	N/R	Outpatients with nonspecific chronic LBP and M1.	Patients with spondylolisthesis associated with M2; did not wear the brace for at least 3 months or did not want to wear one	Wear custom-made rigid lumbar brace all day for 3 months and to take it off if lying down.	N/R
Annen M et al. 2016; SC; Switzerland; 6 months.	40 MCs patients with disc herniation (MC1:16; MC2: 24); mean age: 41.9; 76.4% male; baseline NRS back:5.52; baseline NRS leg:5.71; baseline ODI: 18.88.	32 patients with disc herniation; mean age:38.5; sex distribution: N/R; baseline NRS back: 6.22; baseline NRS leg: 5.44; baseline ODI:15.56.	Age 18–65 years, LBP, and at least one of the following criteria: a) reduced straight leg raise test; b) deficit in detection of cold temperature; c) decreased response to pinprick; d) reduced muscle strength in a corresponding myotome; e) decreased or absent deep tendon reflex corresponding to the involved segment.	Contraindications to chiropractic spinal manipulation, such as tumors, infections, etc; previous spinal surgery; BMI>30; cauda equina syndrome; spondylolisthesis; neurogenic claudication; pregnancy.	High-velocity, low-amplitude spinal manipulation.	
Annen M et al. 2018; SC; Switzerland; 3 months.	57 patients with MCs(MC1:28; M2:29); age: 51.9; sex distribution: N/R; baseline NRS back: 5.50; baseline BQ score: 38.73.	55 patients without MCs; age:37.7; sex distribution: N/R; baseline NRS back: 5.52; baseline BQ score: 42.57.	LBP outpatients without disc herniation; other criteria same as the previous paper by authors.	Same as the paper published in 2016 by authors.	High-velocity, low-amplitude spinal manipulation.	

**Medication**						
Albert HB et al 2008; SC; Denmark; 14 months.	N: 29; age: 45.7 years; 34% female; Patient-specific function scale: 10.5–18.5; pain scale: 6–15; RMQ scale: 4.5–13.5	N/R	MRI displayed M1 in a vertebra adjacent to their previous herniated disc and they had LBP at the time of this follow-up examination.	An allergy to antibiotics, a current infection or declined participation in the antibiotic trial.	Amoxicillin-clavulanate (500 mg/125 mg) three times a day, at 8 h intervals, for 90 days.	N/R
Albert HB et al 2013; RCT; Denmark; 1 year.	N:90; age:44.7 years; 58.2% female; back pain:6.7; leg pain:5.3	N:72; age:45.5 years; 58.2% female; back pain:6.3; leg pain:4.0	Age:18–65 years, MRI confirmed disc herniation within the preceding 6-24 months, LBP of >6 months duration.	Allergy to antibiotics, current pregnancy or lactation, any kidney disease or pending litigation.	amoxicillin–clavulanate (500 mg/125 mg) tablets three times a day, at 8 h intervals, for 100 days.	Placebo
Bråten LCH et al. 2019; RCT; Norway; 1 year.	N:89; age: 44.7 years; 60% female; baseline RMQ score:12.7.	N:91; age: 45.2 years; 57% female; baseline RMQ score:12.8.	Age: 18–65 years; LBP for >6 months with intensity of at least 5 on a 0–10 numerical rating scale; lumbar disc herniation on MRI in the preceding two years; MC1 or MC2 (with height ≥10% of vertebral height and diameter >5 mm) at the herniated disc level.	Had surgery for disc herniation in the past year or antibiotic treatment in thepast month.	3 months oral amoxicillin capsules, mg (3 × daily)	3 months oral maize starch capsules (3 × daily)
Wilkens P et al. 2012; RCT; Norway; 6–18 months.	N:18; age and sex distribution: N/R;	N:24; age and sex distribution: N/R;	Nonspecific chronic LBP for at least 6 months with summed score of at least 3 out of 24 points on the RMQ; > 25 years; with MCs.	Symptomatic disc herniation or spinal stenosis, previous lumbar fracture or surgery, pregnancy or breastfeeding, seafood allergy, ongoing psychiatric or somatic disease potentially influencing a patient's pain, and use of any type of glucosamine 1 year prior to enrollment.	A daily dose of 1500 mg of glucosamine sulfate administered as three 500-mg capsules for 6 months.	A daily dose of 1500 mg of placebo administered as three 500-mg capsules for 6 months.
Koivisto K et al. 2014, 2017; RCT; Finland; 1 year.	N:20; mean age: 49 years; 25% female; median duration of LBP:330 days; mean LBP: 6.6; mean ODI: 30.0.	N:20; mean age: 51; 45% female; median duration of LBP: 315 days; mean LBP: 6.8; mean ODI: 35.0.	LBP for at least 3 months; LBP intensity of at least 6 on a 10-cm VAS or an ODI of at least 30%; The course of MC within 6 months prior to enrolment.	Renal impairment, nerve root entrapment, hypoalcaemia, the presence of red flags, hyper- sensitivity to bisphosphonates or the infusion, willingness for early retirement, and childbearing potential.	A single intravenous infusion of 5 mg ZA in 100 ml saline over a 15-min period	100 ml saline as placebo over a 15-min period.
Zhou JM et al. 2018; CS; China; 3 months	N: 62; age: 53.53 year; 48.39% female; VAS score: 6.25; ODI score: 30.49;	N: 47; age: 52.04 year; 40..43% female; VAS score: 6.34; ODI score: 29.74;	Suffered from LBP more than 3 months and M1 confirmed by lumbar MRI.	Osteoporosis, fracture, tumor, infection, structural deformity or compression of the nerve root; rheumatic or rheumatoid arthritis disease or other serious systemic diseases; prior surgery of lumbar spine.	Intramuscularly injected calcitonin (50 IU) once daily for 4 weeks.	Orally administered diclofenac (75 mg) once daily for 4 weeks.
Shea GKH et al. 2022;RCT; Finland; 6 months	N: 9; age: 59 years; 77.8% female; numerical rating scale: 6.80; ODI score: 32.9	N: 12; age: 54 years; 50% female; numerical rating scale: 6.70; ODI score: 40.5	Suffered from LBP for at least 3 months with a score over past week of at least 5 on a 0–10 numerical rating scale (NRS), or ODI of at least 30%.	Symptoms or signs compatible with nerve root entrapment or spinal stenosis, local or generalized infection, a BMI of >40 kg/m^2^, vertebral fractures, back surgery within 6-months, ect.	50 mg oral ZA once a week for 6 weeks.	Placebo
Jensen OK et al. 2019; RCT; Denmark; 1 year	N: 44; age: 46.1 year; 72.7% female; mean disability score: 14.4; mean back pain score: 6.0; mean leg pain score: 2.6	N: 45; age: 46.3 year; 75.7% female; mean disability score: 13.6; mean back pain score: 5.7; mean leg pain score: 2.7	Age 18–65 ​years; MRI verified MC1 (or mixed MC) within the last 3 ​months; no sign of activation of the immune system at inclusion; back pain dominating over leg pain; back pain duration >3 ​months; moderate disability.	Previous back surgery within the last 6 ​months; planned or treatment by antibiotics for MC within the last 6 ​months; >2 ​weeks antibiotic treatment within the last 3 ​months; autoimmune disease; immune deficiency; malabsorption; cancer or chronic infection.	Probiotic Dicoflor ® twice daily for 100 ​days.Each capsule contains 6 billion Lactobacillus Rhamnosis GG.	Placebo capsules indistinguishable from Dicoflor twice daily for 100 ​days.
**Combined physiotherapy with medication**						
Chen YF et al. 2019; CS; China; 6 months	N: 129 (MC1: 31, MC2: 48); age: 21–67 year; 56.59% female; VAS_M1_ score: 6.4; VAS_M2_ score: 6.3; ODI_M1_ score: 22.0; ODI_M1_ score: 22.7;	N/R	Age with a range of 20–70years, LBP experienced for 3–12 months, without radicular leg pain, and no history of formal treatment.	Mixed MCs, a history of abdominal/pelvic surgery, as well as a specific spinal disease (e.g., scoliosis, spondylolisthesis, infection, and tumor)	Non-surgical treatment for 6 months (two courses) involving the McKenzie method and pharmacological therapy (NSAIDs and muscle relaxants).	

RCT: randomized controlled trial; SC: single-arm observational cohort; CS: comparative observational study; N: number; NRS: numerical rating scale; RMQ: Roland Morris Questionnaire; EQ_VAS_: EuroQol visual analog scale; LBP: low back pain; C: Modic type 1 change; MC2: Modic type 2 change; MC3: Modic type 3 change; MCs: Modic changes; ODI: Oswestry Disability Index; N/R: not report; BMI: body mass index; BQ: Bournemouth Questionnaire; MRI: Magnetic Resonance Imaging; NSAIDs: non-steroidal anti-inflammatory drugs.

**Table 4:** The main results and adverse events reported in each included study.

Study	Intervention	Main reported results	Adverse events
**Physiotherapy**			
Jensen RK et al. 2012	Exercise	1. There were no significant differences between the groups for the outcomes of pain, disability, general health, depression global assessment or the numbers of patients achieving an MCID. 2. Seeking additional care at 1 year follow-up had risen to 30 (64%) in the rest group and 23 (50%) in the exercise group.	No serious problems. Increased pain: 3 cases in the rest group and 5 cases in the exercise group.
Jensen RK et al. 2015	Exercise	1. The effect of rest versus exercise was less in participants with large MCs than in those with small MCs (−1.49, 95%CI −3.73 to 0.75). 2. Patients with M1, or with large MCs, or with large M1 would not benefit more from rest than from exercise.	N/R
Annen M et al. 2016	Spinal manipulation	1. The proportion of patients with MCs or without MCs reporting “improvement” at the different follow-up were quite similar other that at 1 year where there was a tendency for a higher proportion of patients without MCs to report “improvement”. 2. A significant difference in outcomes was noted at 1 year as patients with M2 did significantly better and the proportion of patients with M1 reporting improvement significantly decreased compared to the earlier time points. 3. The Oswestry change scores showed a statistically significant difference, indicating that patients with MCs improved more than patients without MCs.	N/R
Annen M et al. 2018	Spinal manipulation	1. The percentage of patients reporting clinically relevant “improvement” increased in all categories (MC present or absent, M1 or M2) over time. 2. There were no significant difference regarding“improvement” or“worsening” of the patients' reported condition when comparing patients with/without MCs and M1/M2 at any of the collection data time points; 3. There was no significant difference between MCs positive and MCs negative patients nor between M1 and M2 patients for the NRS or BQ change scores at any outcome time point.	N/R
Boutevillain L et al.2018	Rigid lumbar brace	1. Improvement in pain of at least 30% and at least 50% were 79% of patients and 39/62 (62.9%) patients. 2. The mean improvement percentage after 3 months of immobilization with the CRLB was 49%; 3. Pain recurred for 30/46 (65.2%) participants after CRLB withdrawal.	No any adverse events
**Medication**			
Albert HB et al. 2008	Antibiotic	1. All outcome measures (disease-specific function, patient-specific function, global perceived health, and LBP) showed statistically significant improvements both at the end of the treatment period and at the long-term follow-up. 2. Approximately two-thirds of the patients reduced their RMQ scores more than 30%.	Three patients with severe diarrhoea.
Albert HB et al. 2013	Antibiotic	1. Compared to the placebo group, the 1-year improvement was both statistically significant on all outcome measures and clinically important in terms of the relative magnitude of improvement for the primary outcome measures. 2. The percentage of patients with grade 1 Modic changes (minute) 28.8 % of the placebo group and 10.4 % of the antibiotic group were noted, p = 0.006. 3. A significant decrease in volume was observed in the antibiotic group, where changes of volume 2–4 were reduced to volume 1 (p = 0.05).	Adverse events were more common in the antibiotic group (65 %) compared to the placebo group (23 %)
Bråten LCH et al. 2019	Antibiotic	1. At one year, the mean RMQ score had reduced since baseline in both treatment groups (−3.7 points in the amoxicillin group and −2.1 points in the placebo group). 2. The adjusted mean difference in RMQ score between the amoxicillin group and the placebo group at one year was −1.6 points (95% confidence interval −3.1 to 0.0, P = 0.04). 3. The adjusted between group difference of the mean RMDQ score was −2.3 (95% CI −4.2 to −0.4, P = 0.02) for patients with M1 and −0.1 (−2.7 to 2.6, P = 0.95) for patients with M2.	At least one drug related adverse event: 56% (amoxicillin) vs 34% (placebo); serious adverse events: 7% (amoxicillin) vs 2% (placebo).
Wilkens P et al. 2012	Glucosamine sulfate	1. 21 of all 42 patients with pre-treatment MCs had altered MCs type and/or MCs size at follow-up 6–18 months after their initial MRI. 2. Post-treatment, the GS-and placebo group did not differ in proportions of MCs with decreased type 1 dominance (OR 1.6, 95% CI 0.4–6.1; p = 0.46), increased type 1 dominance (OR 2.4, 95% CI 0.6–9.7; p = 0.22), or increased MCs size (OR 1.0, 95% CI 0.2–4.7; p = 0.97).	N/R
Koivisto K et al. 2014	Zoledronic acid	1. The difference in intensity of LBP significantly favoured ZA at one month (MD 1.4; 95% CI 0.01 to 2.9); while at one year no significant difference was observed (MD 0.7; 95%CI-1.0 to 2.4). 2. The proportion of patients with at least 20% improvement in intensity of LBP and PASS both favoured the ZA treatment at one month: ZA 55% vs. placebo 25% (p = 0.105) and ZA 50% vs. placebo 20% (p = 0.096). 3. The improvement in ODI and side bending non-significantly favoured the ZA treatment at one month but not at one year. 4. No differences between the treatment groups at any time point in leg pain intensity, total RAND-36, or in the physical and mental components of RAND-36.	Acute post-infusion phase reactions (fever, headache, myalgia, arthralgia, pain, nausea and flu-like symptoms): 19 (ZA) and 7 (placebo)
Koivisto K et al. 2017	Zoledronic acid	1. The total volume of the primary MCs increased from baseline to 1 year (ZA: 1.6 cm^3^ vs placebo: 2.9 cm^3^; p = 0.21); 2. The change in M1 volume (ZA: −0.83 cm^3^, decrease vs placebo:0.91 cm^3^, increase; p = 0.21); 3. The change in M2 volume (ZA: 1.97 cm^3^, increase vs placebo: 2.40 cm^3^, increase; p = 0.71). 4. The changes in M1 and M2 volumes in relation to intensity of LBP and ODI were weak.	N/R
Shea GKH et al. 2022;	Zoledronic acid	1.In the ZA group, LBP intensity was lower at 4-weeks in comparison to placebo (5.1 ± 1.9 vs. 6.9 ± 1.8, p = 0.038) (minimal clinically important difference [MCID] = 1.5); 2.LBP intensity reduced at 4-weeks and 3-months in the ZA-treated group in comparison to baseline; 3.subscale RAND-36 metrics for physical function (p = 0.038), energy/fatigue (p = 0.040) and pain (p = 0.003) were improved at 3-months compared to placebo, with moderate significant difference for pain at 6-months; 4. A reduction in MC endplate affected area at 6-month follow-up was noted in the ZA group (−0.67 ± 0.69 cm^2^), while in the placebo group no change in size was observed (0.0 ± 0.15; p = 0.041)	3 subjects from the ZA group withdrew due to adverse events. Flu-like symptoms: 1 case; Fever and myalgia: 1 case; Epigastrium discomfort: 1 case.
Zhou JM et al. 2018	Calcitonin	1. Significant improvements were found in VAS and ODI compared with baseline in both groups at 4 weeks and 3 months of follow-up. 2. However, calcitonin group showed a significant difference in VAS, ODI and the proportion of individuals with a significant change (30% reduction compared to baseline) in LBP scales than the diclofenac group (P < 0.05). 3. The proportion of patients with non-M1, a significant improvement on MRI, was 43.54% and 21.27% in the calcitonin and diclofenac groups, (P = 0.015).	17 patients (27.41%) in the calcitonin group and 7 (14.89%) in the diclofenac group (P = 0.118).
Jensen OK et al. 2019	Probiotics	1. No significant differences were detected between the two groups regarding the predefined primary or secondary outcomes, the number of patients reporting global effect of treatment or reporting of minimal disability at 1 ​year. 2. Back pain decreased by 1.1 more (95%CI: 0.20–1.97, p = 0.017) in the active group than in the control group. The corresponding improvement was 22% and 7% in the active and placebo group, respectively, which is less than minimal important change (30%).	No adverse events associated with Probiotic.
**Combined physiotherapy with medication**			
Chen YF et al. 2019	physiotherapy and medication	1. Three months after undergoing nonsurgical treatment, the rates of improved ODI scores were 57.7%, and 48.0%, and those for improved VAS scores were 54.7%, and 46.0%. 2. Again the rates of improved ODI and VAS scores in the MC1 group were 16.1% and 13.8% at 6 months.	N/R

MCID: minimal clinically important difference; MCs: Modic changes; 95%CI: 95%confidence interval; M1: Modic type 1 change; N/R: not report; M2: Modic type 2 change; NRS: numerical rating scale; BQ: Bournemouth Questionnaire; CRLB: custom-made rigid lumbar brace; LBP: low back pain; RMQ: Roland Morris Questionnaire; MRI: Magnetic Resonance Imaging; GS: glucosamine sulfate; OR: odds ratio; ZA: zoledronic acid; MD: mean difference; ODI: Oswestry Disability Index; VAS: visual analog scale.

Of these 15 studies, nine studies [24, 25, 27, 31, 32, 33, 34, 35, 37] were RCTs and the risk of bias results are shown in Table 5. Most RCTs addressed “YES” for adequate the method of randomization (9/9,100%), treatment allocation concealed (8/9, 88.9%), patient blinded to intervention (9/9,100%), outcome assessor blinded to intervention (9/9,100%), drop-out rate described and acceptable (8/9, 88.9%), all randomized participants analyzed in the allocated group (8/9, 88.9%), groups similar at baseline (9/9,100%), cointerventions avoided or similar (8/9, 88.9%), and timing of the outcome assessment similar (9/9,100%). However, a high proportion addressed “UNSURE” for care provider blinded to intervention (6/9, 66.7%), free of suggestion of selective outcome reporting (8/9, 88.9%), compliance acceptable in all groups (7/9, 77.8%), and other sources of potential bias (5/9, 55.6%). Four studies [26, 28, 29, 30] were case series and their methodological qualities were considered as grade IV. The other 2 studies [36, 38] were observational cohort studies and were rated as high quality according to NOQAS with a quality score of more than 6 points. Grades of recommendations for various treatments of MCs are summarized in Table 6.

**Table 5:** The risk of bias results.

Source of Bias	Authors, Year								
	Jensen, 2012	Wilkens, 2012	Albert, 2013	Koivisto, 2014	Jensen, 2015	Koivisto, 2017	Bråten, 2019	Jensen, 2019	Shea, 2022
Adequate the method of randomization	Yes	Yes	Yes	Yes	Yes	Yes	Yes	Yes	Yes
Treatment allocation concealed	Yes	Yes	Yes	Yes	Yes	Yes	Yes	No	Yes
Patient blinded to intervention	Yes	Yes	Yes	Yes	Yes	Yes	Yes	Yes	Yes
Care provider blinded to intervention	Unsure	Unsure	Unsure	Yes	Unsure	Yes	Yes	Unsure	Unsure
Outcome assessor blinded to intervention	Yes	Yes	Yes	Yes	Yes	Yes	Yes	Yes	Yes
Drop-out rate described and acceptable	Yes	Unsure	Yes	Yes	Yes	Yes	Yes	Yes	Yes
All randomized participants analyzed in the allocated group	Yes	Unsure	Yes	Yes	Yes	Yes	Yes	Yes	Yes
Free of suggestion of selective outcome reporting	Unsure	Unsure	Unsure	Unsure	Unsure	Unsure	Yes	Unsure	Unsure
Groups similar at baseline regarding the most important prognostic indicators	Yes	Yes	Yes	Yes	Yes	Yes	Yes	Yes	Yes
Cointerventions avoided or similar	Yes	Yes	No	Yes	Yes	Yes	Yes	Yes	Yes
Compliance acceptable in all groups	Unsure	Unsure	Yes	Unsure	Unsure	Unsure	Unsure	Unsure	Yes
Timing of the outcome assessment similar in all groups	Yes	Yes	Yes	Yes	Yes	Yes	Yes	Yes	Yes
Other sources of potential bias	Unsure	Unsure	Unsure	Yes	Unsure	Yes	Yes	Unsure	Yes

**Table 6:** Grades of recommendations for various treatments of symptomatic MCs.

Treatment options	Clinical questions	Recommendation	NASS Grade
Exercise	Should exercise therapy be used as a conservative treatment for symptomatic MCs?	Conflicting evidence	I
Spinal manipulation	Should spinal manipulation be used as a conservative treatment for symptomatic MCs?	Fair evidence (Suggested)	B
Rigid lumbar brace	Should rigid lumbar brace be used as a conservative treatment for symptomatic MCs?	Insufficient evidence	I
Antibiotic	Should antibiotic be used as a conservative treatment for symptomatic MCs?	Conflicting evidence	I
Glucosamine sulfate	Should glucosamine sulfate be used as a conservative treatment for symptomatic MCs?	Insufficient evidence	I
Zoledronic acid	Should zoledronic acid be used as a conservative treatment for symptomatic MCs?	Fair evidence (Suggested)	B
Calcitonin	Should calcitonin be used as a conservative treatment for symptomatic MCs?	Insufficient evidence	I
Probiotics	Should probiotics be used as a conservative treatment for symptomatic MCs?	Insufficient evidence	I
Physiotherapy and medication	Should combining physiotherapy with medication be used as a conservative treatment for symptomatic MCs?	Insufficient evidence	I

### 3.3 Physiotherapy

#### 3.3.1 Exercise therapy

Two RCTs from Jensen et al. published in 2012 [24] and 2015 [25] reported on the effects of exercise versus rest in CLBP patients with MCs. In the rest group, 49 patients were educated to avoid heavy physical activity and to rest by lying down twice a day for one hour each time. In addition, these patients were also given a flexible belt and instructed to use it for up to four hours a day as required. The remaining 51 patients received exercises for muscle stabilization in the lower back and abdominal together with additional training for postural instability and light physical fitness in a group of up to a maximum of 10 patients under the guidance of a physiotherapist for one hour per week for 10 weeks. These patients were also encouraged to do the same training at home three times a week. Finally, 78 patients completed the full treatment program with a 22% of dropout rate.

In their study published in 2012 [24], no differences were detected in pain, disability, general health, depression, and globe assessment between the two interventions. At the 1-year follow-up, the number of patients seeking additional treatments was 30 (64%) in the rest group while 23 (50%) in the exercise group, without statistical difference. Additionally, there was no significant difference in the increase of pain intensity between the rest (3 cases) and exercise (5 cases) groups.

However, Jensen et al. [25] conducted a secondary subgroup analysis of the above patients in 2015 and found that patients with Modic type 1 change (MC1) were 0.17 points worse in low back pain intensity at rest than exercise (0.17; 95% CI -1.28 to 0.93), those with larger MCs were 0.41 points worse at rest than exercise (0.41; 95% CI -1.62 to 0.79), and those with large MC1 were 0.61 points worse at rest than exercise (0.61; 95% CI - 1.82 to 0.61). Interestingly, this result is contrary to their previous hypothesis that "patients with these MRI findings would benefit more from rest than from exercise therapy".

#### 3.3.2 Rigid lumbar brace

To our best knowledge, only one single-arm observational cohort [30] reported the efficacy of custom-made rigid lumbar brace for the treatment of patients with MC1. Sixty-two CLBP patients with MC1 were asked to wear a custom-made lumbar rigid brace every day (except lying down) for 3 months based on no change in their daily activities. At the final follow-up, the number of patients with pain improvement of at least 30% and 50% was 49 cases and 39 cases respectively, and the mean improvement percentage after 3 months of brace wearing was 49%. However, despite no adverse events, almost all patients experienced a recurrence of pain two months after brace withdrawal.

#### 3.3.3 Spinal manipulation

Two articles by Annen et al. published in 2016 [28] and 2018 [29] were the only two studies that could be retrieved on the efficacy of manipulation in the treatment of patients with CLBP combined with MCs. Both studies used high-velocity, low-amplitude spinal manipulation. The major difference was whether the study subjects had the combined lumbar disc herniation.

A study [28] published in 2016 mainly focused on patients with MCs with lumbar disc herniation. They found that the manipulation therapy had a good short-term effect on CLBP patients with or without MCs. However, the clinical improvement in 76.5% of Modic positive patients and 53.3% of Modic negative patients at 2 weeks, indicated that patients with MCs can benefit more from the manipulation therapy. Moreover, patients with MCs had a larger decrease in the level of disability at 3 and 6 months. And patients with Modic type 2 change (MC2) responded more positively and effectively to the manipulation compared to patients with MC1 (p = 0.001) at 1-year follow-up.

In their subsequent study [29], MCs patients without lumbar disc herniation were employed as study subjects. The result that manipulation therapy was effective in treating CLBP patients with MCs was once again reported. However, there were no significant differences in clinical improvement between Modic positive and negative patients or between MC1 and MC2 patients. Therefore, they concluded that the presence or absence of MCs and the MCs types might be not related to treatment outcomes for MCs patients without disc herniation who underwent chiropractic care.

### 3.4 Medication

#### 3.4.1 Antibiotics

Three studies [26, 31, 32] reported on the safety and efficacy of antibiotics for the treatment of CLBP combined with MCs, two of which were RCTs [31, 32] and another was a single-arm prospective study [26].

In 2008, Albert et al. [26] conducted a prospective study of 32 patients with MCs to first explore the effects of antibiotics on the MCs at the end of Amoxicillin-clavulanate treatment (90 days) and 11-month post-treatment. All clinical indicators improved statistically at follow-up points in the remaining 29 patients, except for 3 patients who withdrew from the study due to severe diarrhea (p < 0.001). The authors, therefore, concluded that this result could provide favorable evidence for the hypothesis of bacterial infection in MCs. Subsequently, they conducted another randomized double-blind trial of 162 CLBP patients with MC1 for a 1-year follow-up [31]. In this study, patients in the antibiotic group received amoxicillin-clavulanate tablets (500mg/125mg) three times a day, 1 or 2 tablets each time, for 100 days. All outcome measures were significantly improved in the antibiotic group and continued to be so at the one-year follow-up. Moreover, there were statistically significant improvements on decreasing the sizes of MCs and clinically important in terms of the relative magnitude of improvement for the primary outcome measures as well as decreasing the sizes of MCs compared to the placebo group. In addition, there was a positive trend towards a dose-response relationship and double-dose antibiotics appeared to be more effective. However, the high rate of adverse events associated with antibiotic treatment was also noted by the authors in the article as such (65% in the antibiotics group vs 23% in the placebo group).

The above conclusions have been overturned by a recent study [32]. In this study, 180 patients with MC1 or MC2 with lumbar disc herniation were randomized to receive oral treatment with either 750 mg amoxicillin or a placebo three times daily for three months. However, these patients did not have significant clinical improvement after three months of amoxicillin treatment compared with the placebo group. Therefore, this study did not support the use of antibiotics in patients with MCs.

#### 3.4.2 Glucosamine sulfate (GS)

Wilkens et al. [33] selected specific subjects from their previous study for subgroup analysis to investigate the effects of GS in the treatment of MCs. 42 patients who completely met the inclusion criteria randomly received GS or placebo 1500mg every day for 6 months. At the treatment endpoint, radiographic parameters revealed that there were no significant differences in proportions of MCs with increased MC1-dominance (OR placebo: GS 2.4, 95% CI 0.6–9.7; p = 0.22), or decreased MC1-dominance (OR GS: placebo 1.6, 95% CI 0.4–6.1; p = 0.46), or increased MC size (OR 1.0, 95% CI 0.2–4.7; p = 0.97). Therefore, the authors concluded that GS has no clear efficacy in CLBP patients with MCs.

#### 3.4.3 Zoledronic acid (ZA)

Three RCTs [27, 34, 35] aimed at investigating the efficacy of ZA in CLBP patients with MCs were included in this systematic review. Two [34, 35] of 3 studies were from a research team and targeted the same subjects, but different indicators were employed to evaluate clinical outcomes after treatments. Forty patients with MCs were divided into two groups to receive either an intravenous infusion of 100 ml saline dissolved in 5 mg ZA (20 patients) or 100 ml of saline as a placebo (20 patients). The remaining one study [27] that included 25 patients with CLBP and MCs (ZA: 13 cases and placebo: 12 cases) was to assess the efficacy of oral 50 mg ZA once a week for 6 weeks.

In 2014, Koivisto et al. [34] reported that patients receiving an intravenous infusion of ZA could obtain a significant benefit in reducing intensity of CLBP at 1 month of treatment (MD 1.4; 95% CI 0.01 to 2.9). However, there were no significant differences in improving the oswestry disability index (ODI) and decreasing CLBP intensity at 1 year of follow-up between ZA and placebo group. Additionally, more mild adverse events that do not require clinical management were observed in the ZA group (ZA: 19/20; placebo: 7/20). In the subsequent study [35], they found that the intensity of CLBP was positively associated with existing MC1, and ZA had a significant effect in reducing the volume of MC1 than placebo (ZA: −0.83 cm^3^, decrease vs placebo: 0.91 cm^3^, increase). However, no significant difference in decreasing the volume of MC2 between both groups was detected (ZA: 2.40 cm^3^, increase vs placebo:1.97 cm^3^, increase). A recent study from Shea et al. [27] has also reached to the similar findings. Patients with oral 50 mg ZA once a week for 6 weeks have significant reduction in MC endplate affected area at 6-month follow-up compared to placebo (p = 0.041). Moreover, subscale RAND-36 metrics for physical function (p = 0.038), energy/fatigue (p = 0.040) and pain (p = 0.003) in the ZA group were improved at 3-months compared to placebo, with significant differences in pain intensity at 4-weeks and 6-months.

#### 3.4.4 Calcitonin

In recent, a study [36] evaluated the efficacy of calcitonin and diclofenac sodium in CLBP patients with MC1. In this retrospective study, 62 patients were injected intramuscularly with calcitonin 50 IU once daily and 47 patients were treated with diclofenac 75 mg once per day for 4 weeks. Compared with baseline, there were significant improvements in visual analogue scale (VAS) and ODI scores at 4-week and 3-month after treatment in both groups (P < 0.05). However, patients treated with calcitonin significantly reduced pain intensity than those with diclofenac. Moreover, 43.54% of patients in the calcitonin group showed improvement on MRI compared to 21.27% in the diclofenac group, with a significant difference between them (P < 0.05).

#### 3.4.5 Probiotics

To investigate whether probiotic is linked to changes in disability and pain in CLBP patients with MC1 or mixed MCs, 89 patients who met inclusion criteria were enrolled in an RCT conducted by Jensen et al. [37] to receive either probiotics capsule (Lactobacillus Rhamnosis GG) or a placebo capsule twice daily for 100 days. The results showed that although back pain decreased by 1.1 more (95% CI: 0.20–1.97, p = 0.017) in the probiotics group than placebo, no statistically significant differences in primary or secondary outcomes between the two groups were detected. Thus, there was no effect of 100-day treatment with probiotics except for a small, almost clinically insignificant effect on back pain caused by MC1 at 1 year.

### 3.5 Combined physiotherapy with medication

Only one study [38] reported on the efficacy of combined physiotherapy with medication in the treatment of patients with MCs. In this study, 129 patients were allocated to three groups according to the presence or absence of MCs and the type of MCs and all received the functional exercise (McKenzie method) in combination with non-steroidal anti-inflammatory drugs (NSAIDs) and muscle relaxants for 6 months. The rates of improvement in ODI scores for CLBP patients with MC1 and MC2 were 57.7% and 48.0%, respectively, and those for VAS scores were 54.7% and 46.0% at three months of treatment. At the treatment endpoint, the ODI and VAS scores for patients with MC1 again improved by 16.1% and 13.8%. However, relevant adverse events did not report in the paper.

## 4 Discussion

MCs are the specific imaging manifestations characterized by CLBP as a clinical feature [39]. The underlying mechanism remains unclear and debated (mechanical, local infection, genetic) [40, 41, 42]. For patients with MCs, the clinical approach remains based on the treatment principles of CLBP, with a focus on reducing pain intensity. As the understanding of MCs has increased, treatment options to address the underlying causes of MCs have been reported but the results are still controversial. To our best knowledge, this is the first systematic review to summarize multiple non-surgical modalities for the treatment of MCs. In this study, we found that 1) The rigid lumbar brace will only improve the patient's clinical symptoms during treatment; 2) Only special subgroups of patients with MCs will benefit in the short term from spinal manipulation (e.g., combined disc herniation) and exercise therapy (e.g., MC1 or larger size of MCs); 3) The rationale and safety of antibiotics for the treatment of MCs remain controversial. 4) ZA and calcitonin have moderate clinical effects in reducing the intensity of CLBP in the short term. However, GS and probiotics have no clear efficacy for such patients.

### 4.1 Non-pharmaceutical treatment

Despite almost all MCs patients accompanied with severe disc degeneration, surgeons rarely pay much more attention to this specific imaging sign. Currently, the mechanism of CLBP caused by MCs remains unclear or uncertain. Patients with MCs are always managed clinically according to the treatment principles of CLBP. Therefore, non-surgical treatments are the most common interventions that patients receive after their first visit.

Non-surgical treatments, such as exercise and spinal manipulation, are as effective as surgery in reducing pain intensity, but at a lower cost and risk of complications [43]. Exercise therapy is widely recommended for the treatment of persistent low back pain [44]. However, given the histological presentation of MCs with microfractures at the endplate, vigorous weight-bearing exercise may inhibit microfracture healing, resulting in MCs patients being less likely to improve with exercise therapy. A study by Jensen et al. [24] published in 2012 confirms this viewpoint, but exercise therapy can still exert a limited clinical effect especially in specific populations such as those with larger MC1.

Patients with MCs often suffer from lumbar instability [16] and the mechanical receptors on the endplate are stimulated during movement to produce pain. The custom-made rigid brace can provide immediate lumbar support and is an acceptable treatment option for patients with MCs [45]. However, it is predictable that its efficacy should not last too long, which is related to the fact that it only provides temporary lumbar support. Spinal manipulation is also a well-established method of managing CLBP. Although the two studies from Annen et al. [28, 29] targeted on the different study populations, similar findings suggest that spinal manipulation might be considered as an alternative therapy for patients with MCs.

There are no international guidelines for either exercise or manipulative therapy in the treatment of MCs. Generic or inappropriate physiotherapy prescriptions may be effective for only specific patients, which explains the inconsistent results reported across studies. People with CLBP present as a heterogeneous population which highlights the need to provide individualized treatment approaches. Individual studies using specific treatments are difficult to provide strong evidence for clinical practice, and physiotherapy for MCs is still in the exploratory phase and needs further validation. As such, the present work does not yet provide clear guidance to clinicians in their decision-making.

### 4.2 Pharmaceutical treatment

Low-toxicity anaerobic bacteria can continue to grow and spread to the endplates and their adjacent bone marrow after reaching the intervertebral discs through the blood circulation [46], leading to the occurrence of MCs. This result has been confirmed by previous animal experiments [47]. Considering that one hypothesis is that bacterial infection might be a potential mechanism, antibiotics were adopted experimentally to treat MCs in several studies. Both studies performed by Albert et al. [26, 31] revealed that patients with MCs could obtain clinical improvement after antibiotics treatment but with high adverse events. To verify the reliability of the above findings, a replicated randomized multicentre study was recently published in the journal of BMJ [32]. However, the findings from this study have aroused widespread interest and intense debate in academia about the rationale for the antibiotic treatment of MCs.

Although positive bacterial culture results from disc tissue have been reported in several studies [48, 49], the similarity of these positive bacteria to colonies existing skin or muscle still cannot rule out the possibility of contamination from other tissues adjacent to the surgical area. Previous studies have not been able to clarify whether MCs are infectious and therefore the use of antibiotics to treat MCs may not be justified. Additionally, the rational use of antibiotics must involve the selection of the right drug, the appropriate dose and duration, and these clinical issues still need to be explored in subsequent high-quality, multicentre studies. Moreover, antibiotic treatment for a large population may also increase the risk of antibiotic resistance [18], which needs to be considered carefully in clinical practice. Instead, the priority should be to clarify the relationship between bacterial infections and MCs, which may be more important than exploring the safety and efficacy of antibiotics in the treatment of such patients.

ZA is a potent bisphosphonate that inhibits osteoblast recruitment, differentiation and function, and promotes apoptosis [50]. Moreover, calcitonin is a potent inhibitor of bone resorption in osteoblasts, primarily for the treatment of osteoporosis and other diseases involving high bone turnover [51]. Considering their pharmacological properties, they have been introduced experimentally in the treatment of MCs. The two studies [34, 35] included in the present work that explored ZA and calcitonin for patients with MCs both obtained promising results in the short term.

The pathological process of MCs mainly involves inflammation, high bone turnover, and fibrosis [52]. MC1, which is closely associated with inflammation, is thought to be highly linked to pain, while MC2 and MC3 are reported to be less painful [41]. Not only is the occurrence of CLBP correlated with the stimulation of mechanoreceptors caused by endplate microfracture, but it also leads to the stimulation of chemoreceptors by the release of pro-inflammatory factors [35]. The pharmacological effects of calcitonin in maintaining subchondral and trabecular microstructure and promoting the cartilaginous phase of fracture healing reported in relevant animal studies [53] may be able to explain the findings of Zhou et al. [36] In contrast, ZA, a type of bisphosphonates, inhibits the secretion of pro-inflammatory cytokines such as interleukin 1 (IL-1), TNF-α, and IL-6 [54] and reduces bone marrow edema on MRI [55]. It can be assumed that it exerts its clinical effects by interfering with the pathological process of MCs and accelerating the conversion of MC1 to MC2. However, the pain-causing mechanism of MCs may have consisted of multiple factors or cytokines. Despite the ability of GS to slow the destruction of osteoarthritic cartilage by inhibiting IL-1β [56], patients with MCs failed to benefit from it. This may be able to suggest that IL-1β has little to do with the pathology of MCs or that GS cannot reach the target area due to inadequate blood supply to intervertebral discs and vertebral bodies [33]. Clinical interventions that target the pathological process of MCs may provide new ideas for the treatment of such patients. However, we should still pay much more attention to the adverse events of these drugs, especially in patients with hepatic or renal dysfunction.

### 4.3 Limitations

Similar to other studies, some limitations of the present work should be pointed out. First, present work included multiple managements in the treatment of MCs. However, the results of most interventions were only reported by individual studies, which may reduce the strength of the evidence. Moreover, although we attempt to focus on participants with MCs, it's still unable to control all potential sources of variability among participants. Additionally, due to the different incidence among three types of MCs, most studies have not been able to analyze them separately. In contrast, each of these three types represents a different stage in the pathological process of MCs and they may respond differently to treatment options, allowing for heterogeneity in study results. Finally, this study suffers from publication bias and language bias, as we only included the published paper written in English. Although the exclusion of non-English articles does not usually have a significant impact on the results of systematic reviews [57], we were unable to determine this definitively.

## 5 Conclusions

There were several non-surgical interventions used to treat CLBP patients with MCs, with the aim of reducing pain intensity. This systematic review of 15 studies involving MCs patients with clinical results provides limited evidence that patients treated with ZA and calcitonin can achieve short-term symptomatic improvement. However, current findings don't suggest that GS and Probiotics are effective in the treatment of MCs. Exercise and manipulative therapy may only work well for certain patients with MCs. In contrast, the rationale of antibiotic treatment for MCs has not been proven. To sum up, there is not yet enough evidence to suggest that non-surgical treatments are useful for patients with MCs. Further high-quality, multicenter trials are required to validate the effectiveness of these non-surgical treatments.

## Declarations

### Author contribution statement

Xiaoping Mu: Conceived and designed the experiments; Performed the experiments; Wrote the paper.

Jianxun Wei: Conceived and designed the experiments; Contributed reagents, materials, analysis tools or data.

Wei Peng: Performed the experiments; Analyzed and interpreted the data; Wrote the paper.

Yufu Ou: Performed the experiments; Contributed reagents, materials, analysis tools or data.

Peifeng Li: Analyzed and interpreted the data; Contributed reagents, materials, analysis tools or data.

Zhuhai Li: Analyzed and interpreted the data.

### Funding statement

Xiaoping Mu was supported by Scientific Research and Technology Development Program of Guangxi [AD22035004].

Jianxun Wei was supported by Guangxi Natural Science Foundation [2016GXNSFAA380058].

Yufu Ou was supported by Guangxi Natural Science Foundation [2020GXNSFAA297217].

Zhuhai Li was supported by Scientific Research Project of Guangxi Health and Family Planning Commission [Z20210722].

### Data availability statement

Data included in article/supp. material/referenced in article.

### Declaration of interest's statement

The authors declare no conflict of interest.

### Additional information

No additional information is available for this paper.
